# Supplementary material for: AI-Predicted mTOR Inhibitor Reduces Cancer Cell Proliferation and Extends the Lifespan of C. elegans
Source: Int J Mol Sci. 2023 Apr 25;24(9):7850. doi: 10.3390/ijms24097850 (PMC10177929; doi:10.3390/ijms24097850)
Supplement: Supplementary file 1 [file ijms-24-07850-s001.zip › Supplementary Figures S1-S3.pdf]

Supplementary Figures S1-S3

AI-predicted mTOR inhibitor reduces cancer cell proliferation and extends the lifespan of *C. elegans*

Tinka Vidovic, Alexander Dakhovnik, Oleksii Hrabovskyi, Michael R. MacArthur, Collin Y. Ewald

Medicinal Chemistry

QED	0.859
SAscore	2.509
Fsp3	0.421
MCE-18	48.889
NPscore	-1.856
Lipinski Rule	Accepted
Pfizer Rule	Accepted
GSK Rule	Accepted
Golden Triangle	Accepted
PAINS	0 alert(s)
ALARM NMR Rule	0 alert(s)
BMS Rule	0 alert(s)
Chelator Rule	0 alert(s)

Absorption

Caco-2 Permeability	-5.408
MDCK Permeability	1.70E-05
Pgp-inhibitor	+
Pgp-substrate	+++
HIA	---
F20%	---
F30%	---

Distribution

Plasma Protein Binding	93.08%
Volume Distribution	0.702
BBB Penetration	--
Fu (fraction unbound in plasms)	6.46%

Metabolism

CYP1A2 inhibitor	++
CYP1A2 substrate	+
CYP2C19 inhibitor	--
CYP2C19 substrate	--
CYP2C9 inhibitor	---
CYP2C9 substrate	--
CYP2D6 inhibitor	--
CYP2D6 substrate	++
CYP3A4 inhibitor	-
CYP3A4 substrate	-

Excretion

CL	5.107
T1/2	0.546

Tox21 Pathway

NR-AR	---
NR-AR-LBD	---
NR-AhR	++
NR-Aromatase	---
NR-ER	-
NR-ER-LBD	---
NR-PPAR-gamma	---
SR-ARE	+
SR-ATAD5	---
SR-HSE	---
SR-MMP	-
SR-p53	+

Physicochemical Property

MW	369.18
Volume	365.854
Density	1.009
nHA	8
nHD	2
nRot	6
nRing	4
MaxRing	9
nHet	8
fChar	0
nRig	23
Flexibility	0.261
Stereo Centers	0
TPSA	88.61
logS	-4.606
logP	1.817
logD	2.583

Supplementary Figure S1. ADMET of TKA001. The SMILES string of TKA001 was used to obtain the prediction of ADMET properties using the ADMETlab 2.0 tool. QED is a measure of drug-likeness based on the concept of desirability (attractive: > 0.67; unattractive: 0.49~67; too complex: < 0.34). SAscore is the synthetic accessibility score and is designed to estimate the ease of synthesis of drug-like molecules (SAscore > 6, difficult to synthesize. SAscore < 6, easy to synthesize). Fsp3 is the number of sp3 hybridized carbons / total carbon count, and it correlates with melting point and solubility (Fsp3 > 0.42 is considered a suitable value). MCE-18 stands for medicinal chemistry evolution (MCE-18 > 45 is considered a suitable value). NP is a Natural product-likeness score. This score is typically in the range from -5 to 5; the higher the score, the higher the probability that the molecule is an NP. Lipinski rule: MW < 500; logP < 5; Hacc < 10. Hdon < 5, if two properties are out of range, poor absorption or permeability is possible, and one is acceptable. Pfizer rule: logP > 3. TPSA < 75. Compounds with a high log P (>3) and low TPSA (<75) are likely to be toxic. GSK rule: MW < 400. logP < 4. Compounds satisfying the GSK rule may have a more favorable ADMET profile. PAINS - Pan Assay Interference Compounds, frequent hitters, alpha-screen artifacts, and reactive compounds. ALARM NMR - Thiol reactive compounds. BMS - Undesirable, reactive compounds. Chelator rule: chelating compounds. Caco-2 permeability - Optimal: higher than -5.15 Log unit. MDCK permeability - low permeability: < 2 × 10-6 cm/s; medium permeability: 2–20 × 10-6 cm/s; high passive permeability > 20 × 10-6 cm/s. Pgp-inhibitor - the output value is the probability of being a Pgp-inhibitor. Pgp-substrate - The output value is the probability of being Pgp-substrate. HIA - Human Intestinal Absorption; Category 1: HIA+ (HIA < 30%). Category 0: HIA- (HIA > 30%). The output value is the probability of being HIA+. F20% - 20% Bioavailability. Category 1: F20%+ (bioavailability < 20%). Category 0: F20%- (bioavailability > 20%). The output value is the probability of being F20%+. F30% - 30% Bioavailability. Category 1: F30%+ (bioavailability < 30%). Category 0: F30%- (bioavailability > 30%). The output value is the probability of being F30%+. PPB - Plasma Protein Binding (Optimal: < 90%. Drugs with high protein-bound may have a low therapeutic index). VD - Volume Distribution. Optimal: 0.04-20L/kg. BBB - Blood-Brain Barrier Penetration. Category 1: BBB+. Category 0: BBB-. The output value is the probability of being BBB+. Fu - The fraction unbound in plasms. Low: < 5%. Middle: 5~20%. High: > 20%. CL – Clearance. High: >15 mL/min/kg. Moderate: 5-15 mL/min/kg. Low: <5 mL/min/kg. T1/2 - Category 1: long half-life; Category 0: short half-life; long half-life: >3h; short half-life: <3h. The output value is the probability of having a long half-life. The prediction probability values are transformed into six symbols: 0-0.1(---), 0.1-0.3(--), 0.3-0.5(-), 0.5-0.7(+), 0.7-0.9(++), and 0.9-1.0(+++).

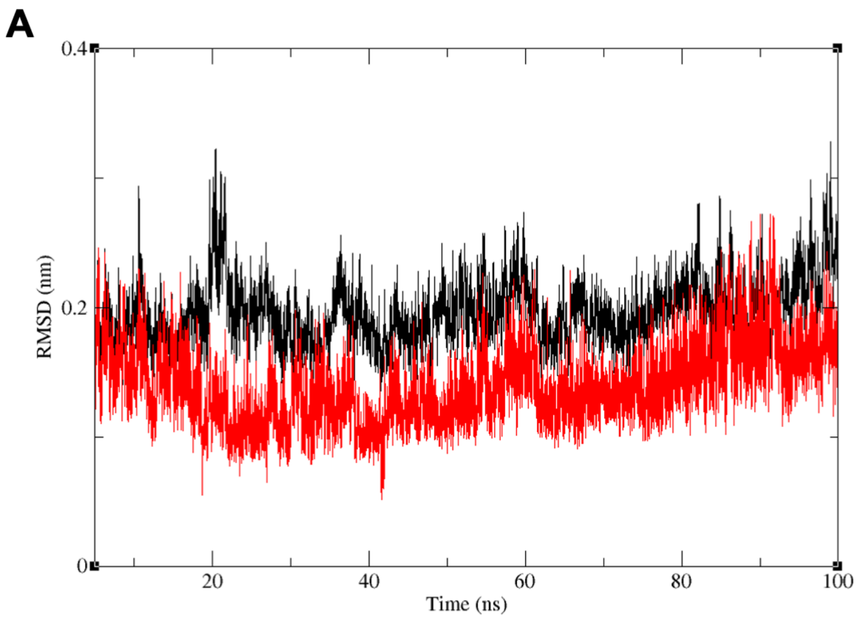

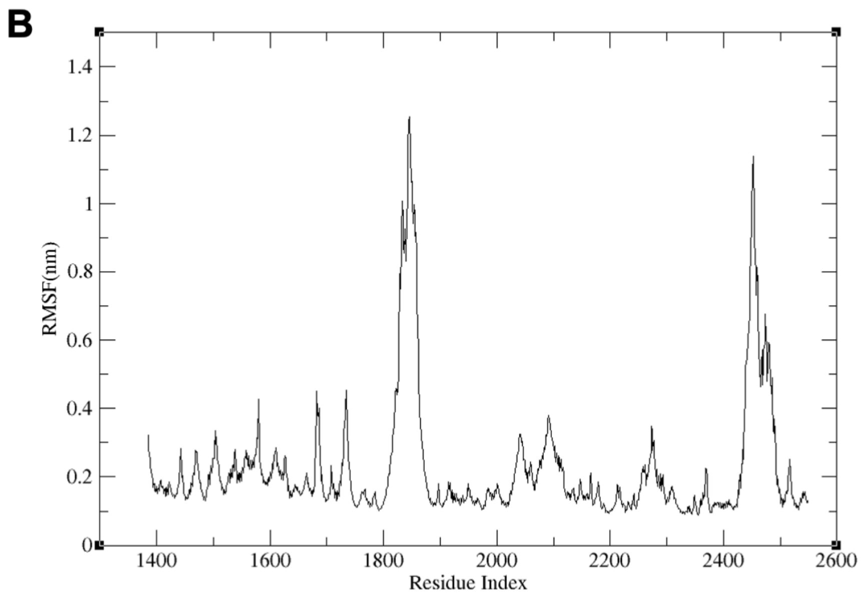

Supplementary Figure S2. Molecular Dynamics of TKA001 with mTOR kinase. A) Root-Mean-Square Deviation (RMSD) values extracted from 100 ns molecular dynamics simulation. RMSD of protein backbone (in black line) and RMSD of TKA001 ligands heavy atoms (in red line). B) Root mean square fluctuations (RMSF) values extracted from protein Cα atoms during 100 ns molecular dynamics simulation.

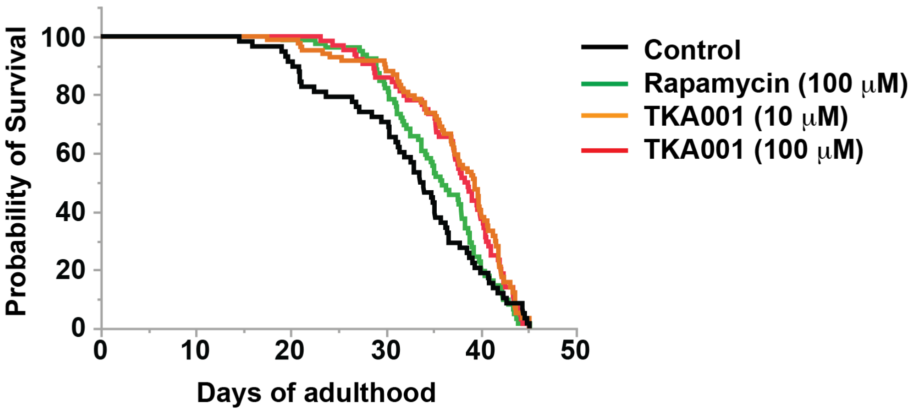

Supplementary Figure S3. Automated lifespan analysis of mTOR inhibitor TKA001. Adult wild-type (N2) *C. elegans* were supplemented with 10, 100 μM TKA001, and 100 μM rapamycin (as a positive control for mTOR inhibition), which increased the mean lifespan of *C. elegans* compared to 0.2% DMSO-treated control. The survival was scored by the automated lifespan machine. Raw data and statistics are presented in Supplementary Table S2.
